# Targeted Central Nervous System Irradiation with Proton Microbeam Induces Mitochondrial Changes in *Caenorhabditis elegans*

**DOI:** 10.3390/biology12060839

**Published:** 2023-06-09

**Authors:** Ahmad Sleiman, Kévin Lalanne, François Vianna, Yann Perrot, Myriam Richaud, Tanima SenGupta, Mikaël Cardot-Martin, Pascal Pedini, Christophe Picard, Hilde Nilsen, Simon Galas, Christelle Adam-Guillermin

**Affiliations:** 1 Institut de Radioprotection et de Sûreté Nucléaire, IRSN, PSE-SANTE/SDOS/LMDN, Cadarache, 13115 Saint-Paul-lez-Durance, France; ahmad.sleiman@irsn.fr (A.S.);; 2 Institut de Radioprotection et de Sûreté Nucléaire, IRSN, PSE-SANTE/SDOS/LDRI, 92262 Fontenay-aux-Roses, France; 3 IBMM, University of Montpellier, CNRS, ENSCM, 34093 Montpellier, France; 4 Section of Clinical Molecular Biology (EpiGen), Akershus University Hospital, 1478 Lørenskog, Norway; 5 Aix Marseille University, CNRS, EFS, ADES, 13288 Marseille, France; 6 Department of Microbiology, Oslo University Hospital, 0372 Oslo, Norway

**Keywords:** *C. elegans*, nervous system, proton, ion microbeam, mitochondria, proton therapy

## Abstract

**Simple Summary:**

Radiotherapy is a common treatment for cancer and is used for approximately half of cancer patients around the globe. In recent years, significant advancements in technology and imaging have allowed for more accurate targeting of tumor cells using protons while minimizing damage to healthy tissues. Despite these advancements, the complete eradication of treatment-related complications for patients remains an ongoing challenge. In this context, research studies are being conducted on the biological mechanisms involved in the initiation and progression of these side-effects to quantify their risk of occurrence and to offer new therapies for treating them. Using the nematode *Caenorhabditis elegans* biological model, the consequences of targeted central nervous system proton irradiation were studied. *C. elegans* were micro-irradiated with 220 Gy of protons (4 MeV) in the central nervous system and the mitochondrial function was assessed. Our results indicate that proton irradiation induced the instant loss of mitochondrial membrane potential in the targeted area with oxidative stress and an increase in the mitochondrial DNA copy number 24 h after irradiation. Furthermore, proton irradiation induced autophagy in the targeted region. This study shows the global mitochondrial damage in the central nervous system area following proton exposure. These results highlight the important role of mitochondria in radiation-induced damage in healthy tissues.

**Abstract:**

Fifty percent of all patients with cancer worldwide require radiotherapy. In the case of brain tumors, despite the improvement in the precision of radiation delivery with proton therapy, studies have shown structural and functional changes in the brains of treated patients with protons. The molecular pathways involved in generating these effects are not completely understood. In this context, we analyzed the impact of proton exposure in the central nervous system area of *Caenorhabditis elegans* with a focus on mitochondrial function, which is potentially implicated in the occurrence of radiation-induced damage. To achieve this objective, the nematode *C. elegans* were micro-irradiated with 220 Gy of protons (4 MeV) in the nerve ring (head region) using the proton microbeam, MIRCOM. Our results show that protons induce mitochondrial dysfunction, characterized by an immediate dose-dependent loss of the mitochondrial membrane potential (ΔΨm) associated with oxidative stress 24 h after irradiation, which is itself characterized by the induction of the antioxidant proteins in the targeted region, observed using SOD-1::GFP and SOD-3::GFP strains. Moreover, we demonstrated a two-fold increase in the mtDNA copy number in the targeted region 24 h after irradiation. In addition, using the GFP::LGG-1 strain, an induction of autophagy in the irradiated region was observed 6 h following the irradiation, which is associated with the up-regulation of the gene expression of *pink-1* (PTEN-induced kinase) and *pdr-1* (*C. elegans* parkin homolog). Furthermore, our data showed that micro-irradiation of the nerve ring region did not impact the whole-body oxygen consumption 24 h following the irradiation. These results indicate a global mitochondrial dysfunction in the irradiated region following proton exposure. This provides a better understanding of the molecular pathways involved in radiation-induced side effects and may help in finding new therapies.

## 1. Introduction

Radiotherapy is an essential modality in the therapeutic arsenal against cancer. Epidemiological data show that almost 50% of people affected by cancer will receive at least one round of radiotherapy as part of their treatment [1]. It is anticipated that cancer rates will reach 26 million new cases by 2030, an increase from approximately 9 million in 2017 [2]. Therefore, the number of patients receiving radiotherapy worldwide every year has increased. However, the use of radiation is not trivial. When it interacts with cells, radiation damages biomolecules such as DNA and proteins, induces oxidative stress and modifies mitochondrial activity by influencing the formation of ROS and ATP levels [3,4]. Malignancies in the central nervous system is one of the cancer types for which radiotherapy remains a mainstay of tumor management. Thus, cerebral radiation therapy can trigger central nervous system complications, raising the issue of the acute and delayed toxicity of the treatments. Radiation-induced cognitive impairment is a common late effect of radiation therapy, seen in more than 30% of patients alive at 4 months after partial or whole brain irradiation. For those who live for over 6 months, that number may rise to 50–90% according to some reports [5]. Most of the available data are inferred from photon therapy and demonstrate that ionizing radiation (IR) induces changes in the neuronal structure and function after irradiation [6,7], as well as a cognitive decline [8]. Patients with cognitive decline struggle to continue working or to live independently as the impairment is marked by decreased verbal and spatial memory, attention and problem-solving ability, with incidence and severity increasing over time [5]. Over the past few decades, technical advances in imaging have enabled the more precise irradiation of tumors, leading to a reduction in the volume of normal tissue irradiated, thanks to hadron therapy, which relies on the use of particles in radiotherapy (mostly protons and carbon ions). The technique came into focus due to the absorption profile in the tissue with lower entrance doses and maximum dose deposition at the end of their range, called the Bragg peak. It thus enables the precise irradiation of deep-seated tumors while sparing the surrounding healthy tissue [9,10,11]. However, the risk of sequelae after treatment for patients has not yet been completely eliminated. In the case of brain tumors, despite the improvement in the precision of radiation delivery with hadron therapy, studies have shown the modification of brain function and structure after irradiation with protons in patients, with a high incidence of necrosis [12]. Moreover, recent studies from animal models reported persistent changes in the neuronal structure and synaptic plasticity caused by proton irradiation [13,14,15]. The majority of the existing findings were mostly deduced through anatomical changes such as vascular abnormalities, demyelination and, ultimately, white matter necrosis. However, in recent years, there has been a growing appreciation that patients receiving irradiation can develop significant cognitive impairment, even in the absence of detectable anatomic abnormalities, thus highlighting the importance of the alterations induced by radiation at the molecular and cellular levels.

Considering the continuously improving radiation treatments and the expected surge in the use of proton therapy, the number of patients with a longer lifespan and, subsequently, a higher risk for developing treatment-related late effects will increase. Therefore, in light of the limited available data and sparse studies on the impact of protons on the central nervous system (SNC), there is a pressing need to investigate the molecular pathways involved in the generation of the radio-induced neuronal damages after proton exposure in the SNC area. At the nuclear level, the DNA is considered as a primary target of radiation [16]. At the cytoplasmic level, a wide range of studies have proven the potential of ionizing radiation to alter the mitochondrial function and structure in different biological models and tissues, as mitochondria occupy around 30% of the cellular volume [17,18]. In addition, recent radiation research revealed that brain irradiation induces changes in mitochondrial activity by influencing the formation of ROS and ATP levels, which leads to brain dysfunction and declines in spatial memory [3].

In this study, we report the first results obtained following analysis of the impact of protons on mitochondrial function in the SNC region of *C. elegans*. The nematode *C. elegans* were micro-irradiated with 220 Gy of protons (4 MeV) in the head region, where the nerve ring resides, where a concentration of neurons wraps around the pharynx, using the ion microbeam of the MIRCOM facility [19]. *C. elegans* are used as a model organism because their genome is fully sequenced, their body is transparent and their internal structure can be observed. In radiobiology, the effects of radiation on vital functions, such as development, locomotion, learning and aging, have been described in *C. elegans* [20,21]. Our data show a global mitochondrial damage characterized by the immediate loss of mitochondrial membrane potential, the induction of cytoplasmic and mitochondrial oxidative stress, an increase in the mtDNA copy number and increased autophagy in the irradiated region. However, the whole-body oxygen consumption rate, determined using a Seahorse XF^e96^ Analyzer, was not significantly changed by targeted irradiation of the nerve ring area.

## 2. Materials and Methods

### 2.1. C. elegans Strain and Culture Conditions

The *sod-1*::GFP transgene, (GA508 wuIs54[pPD95.77 *sod-1::*GFP, *rol-6(su1006)*] was a gift from Prof. David Gems from the Institute of Healthy Ageing Genetics, University College London. The strains Bristol N2; CF1553 [muIs84 [(pAD76) *sod-3p::*GFP + *rol-6(su1006)*] and DA2123 adIs2122 [*lgg-1p*::GFP::lgg-1 + *rol-6(su1006)*] were kindly provided by the Caenorhabditis Genetic Centre (University of Minnesota, St Paul, MN, USA). Worms were cultured and manipulated using previously described methods. Briefly, they were cultured on growth medium (NGM) agar on Petri plates supplemented with *E. coli* (OP50) as a food source [22]. The worms were continuously fed for many generations and maintained at 20 °C in a temperature-controlled incubator.

### 2.2. Site Specific Microbeam Irradiation

Synchronized young adult *C. elegans* hermaphrodites were exposed individually to 4 MeV protons using the ion microbeam of the MIRCOM facility. This facility is an irradiation platform with an ion microbeam capable, with micrometric precision, of targeting cellular or subcellular elements with a defined number of charged particles and with a beam diameter on the sample of 2.2 ± 0.3 μm for protons [19].

The specific dish for worm irradiation consisted of a Polyether ether ketone (PEEK) disc (Lorton, France) serving as a base for the culture well and a ring (Lorton, France) for the irradiation well. Between the ring and the base of the well, a 4 µm thick polypropylene sheet (Goodfellow, UK) was placed (facing the microbeam). The nematodes, anesthetized with 5 mM levamisole, were seeded immediately before irradiation (at least 10 adults in 20 μL of M9, using sterile platin spatula). A drop of 2% agarose gel was then added and a 9 mm coverslip was used to evenly spread the gel layer to ensure improved microscopic observation. During exposure, the worms were individually imaged using an epifluorescence microscope (AxioObserver™ Z1, Carl Zeiss Microscopy GmbH, Jena, Germany) equipped with a Colibri2™ LED light source (Carl Zeiss Microscopy GmbH). The worms were exposed to a given number of protons, defined by a beam opening time—as described in Vianna et al., 2022—covering a region of 50 μm in the head. Non-irradiated control worms were always in the same set-up time for anesthetic exposure and concentrations. After exposure, the coverslips and polypropylene sheet were removed and the worms were washed with M9 buffer and re-cultured in standard agar-covered Petri dishes. All steps were performed at room temperature (22 ± 2 °C).

### 2.3. Monitoring In Vivo Reactive Oxygen Species (ROS) Production Response to Ionizing Radiation in C. elegans

When using microbeam-assisted microscopy, it is important to monitor the fluorescence signal in a delimited region of interest. While conventional redox-sensitive fluorogenic probes are often nonspecific, irreversible and disruptive, and will label the whole body, genetically encoded fluorescent sensors can overcome such limitations and show the fluorescence expression in the targeted area. Therefore, in the current study, the *sod-1*::GFP and *sod-3*::GFP reporter strains were employed as in vivo proxies for ROS production following high-dose proton irradiation. Specifically, the *sod-1*::GFP reporter strain was implemented to measure the expression of the cytosolic and mitochondrial superoxide dismutase 1, SOD-1, while the *sod-3*::GFP reporter strain measures the mitochondrial expression of SOD-3. The fluorescence signals were quantified in the irradiated region. These strains were treated before irradiation with Paraquat, H_2_O_2_ and menadione as positive controls for method validation.

### 2.4. Mitochondrial Membrane Potential Imaging Using TMRE Staining

Tetramethylrhodamine ethyl ester, perchlorate (TMRE) is a dye that accumulates in intact, functional mitochondria. Adult animals were grown at 20 °C in the presence of 1 µM TMRE for 24 h. Stained and washed worms were immobilized with levamisole before being mounted on 2% agarose pads for microscopic examination with epifluorescence microscopy (AxioObserver™ Z1, Carl Zeiss Microscopy GmbH), equipped with the source Colibri2™ LED (Carl Zeiss Microscopy GmbH). The visualization of the samples was performed using an AxioCam™ monochromatic camera (Carl Zeiss Microscopy GmbH). Images were acquired under the same exposure conditions for each animal. Average pixel intensity values were calculated for each worm at t = 0 s and t = 180 s after irradiation in the targeted region. We calculated the relative change in the pixel intensity for each animal using the Fiji Software and the average of the relative change in each group was calculated. Three independent groups of 8 animals (total, *n* = 24) were irradiated with 2700 protons and three independent groups of 5 animals (total, *n* = 15) were irradiated with 10,000 protons. 

### 2.5. Measurement of Mitochondrial DNA Copy Number

As a measure of mitochondrial health, the relative mitochondrial DNA copy number was measured in the worm samples through qPCR and ddPCR.

- DNA extraction:

DNA extraction from *C. elegans* for qPCR and ddPCR was performed using a Sigma Extract-N Amp kit (catalog # XNAT2 kit). The instructions provided by the manufacturer were modified by Madhu et al. and published in the wormbook [23]. Briefly, at the desired time, the animals in each treatment group were individually picked on a microscope slide with droplets of lysis buffer (2 μL of extraction solution and 0.5 μL of Tissue Preparation Solution provided in the kit). Using a 23 G needle, the heads were cut on a microscope slide under the stereomicroscope and 10 heads were picked in 2 μL of lysis buffer and instantly placed on ice in 0.2 mL sterile PCR tubes. The tubes were centrifuged briefly (5 s) and then put in the thermocycler at 55 °C for 10 min, then 95 °C for 3 min. When the program was complete, the tubes were briefly centrifuged and 2 µL of neutralization solution was added to the mixture and mixed by pipetting. The extract can be used for qPCR directly or can be stored at −20 °C for future use. PCR reaction was possible with as little as one single head, but a suspension of 10 heads per 2 microliters yielded the most stable and consistent condition for our experiments (i.e., less variability in the Ct values between samples).

- by qPCR:

Before running the qPCR, 20 μL of nuclease free water was added to the DNA extract after two cycles of freeze thaw for 5 min each. The PCR primers used were *cox-4* for nuclear DNA (reference for normalization) (forward: GCCGACTGGAAGAACTTGTC and reverse: GCGATCACCTTCCAGTA) and *nd-1* for mitochondrial DNA (forward: AGCGTCATTTATTGGGAAGAC and reverse: AAGCTTGTGCTAATCCCATAAATGT). The relative change of mitochondrial DNA was calculated using 2^−ΔΔCt^ method [24]. The qPCR experiments were run using the Applied Biosystems 7500 Real-Time PCR System with Brilliant III Ultra-Fast SYBR Green High ROX qPCR Master Mix (Agilent, Santa Clara, CA, USA, catalog#600889) in two steps: 10 min at 95 °C, followed by 40 PCR cycles (95 °C, 15 s and 60 °C, 60 s).

- by ddPCR

Despite the fact that qPCR is a widely accepted method to measure mtDNA variation levels, the advantages of ddPCR vs. qPCR have been discussed [25]. Here, we confirmed the qPCR results with ddPCR. The mitochondrial DNA (mtDNA) copy number was quantified using droplet digital PCR (ddPCR). The lysates extracted using the same protocol as described above were further diluted and assayed using the Droplet Digital PCR QX system (Bio-Rad, Hercules, CA, USA). Briefly, 10 μL of the diluted lysate was added to a PCR mixture containing 2x QX200 ddPCR EvaGreen Supermix (Bio-Rad) and 600 nM *nd-1* primers and *cox-4*. Then, 40 μL of droplets were generated using a QX100 Droplet Generator (Bio-Rad) by mixing the 20 μL of PCR mixture and 70 μL Droplet generation oil for EvaGreen (Bio-Rad). As described by SenGupta et al., (2021), the following PCR conditions were used after denaturing at 95 °C for 5 min: 40 cycles at 95 °C for 30 s and 60 °C for 1 min were followed by 1 step at 4 °C for 5 min and 90 °C for 5 min. The cycled droplets were read in the QX200 Droplet Reader (Bio-Rad) and analyzed using the QuantaSoft droplet reader software.

### 2.6. Single Worm Gene Expression Analysis

The transcriptional activation of several genes was measured in age-synchronized N2. Similarly to the method described above, at the chosen sampling time, 10 heads were collected in 2 μL PBS and immediately snap frozen in dry ice. For *sod-1*, *sod-3*, *hmg-5* and *polg-1*, mRNA samples were extracted from the heads 24 h after irradiation. For the *pink-1* and *pdr-1* genes, mRNA samples were extracted 5 h after irradiation. cDNA synthesis was performed using a Maxima H Minus First Strand cDNA Synthesis Kit, with dsDNase, as described by [26]. Quantitative reverse transcriptase PCR (qRT-PCR) was performed using a Brilliant III Ultra-Fast SYBR Green High ROX qPCR Master Mix (Agilent, catalog#600889). Gene’s transcript levels were normalized to *act-1*, *pmp-3* or *Y45F10D.4* as housekeeping genes. Each sample was deposited in duplicate in 96-well plates, and the mean of CT was used to calculate the relative fold change of the amplified product using the 2^−ΔΔCt^ method [24]. The qPCR experiments were run in two steps: 10 min at 95 °C followed by 40 PCR cycles (95 °C, 15 s and 60 °C, 60 s) using the Applied Biosystems 7500 Real-Time PCR System (Applied biosystems, Waltham, MA, USA). Three independent experiments were performed, each consisting of three independent groups (10 heads per group; *n* = 90). The average fold change was calculated for each group and the mean of fold change of the three experiments is shown.

### 2.7. Analysis of Fluorescence Intensity

In the transgenic strain of DA2123, *sod-1*::GFP and CF1553, LGG-1::GFP, SOD-1::GFP and SOD-3::GFP are, respectively, expressed in all cells of nematodes. After exposure, the expression level was assayed through the fluorescence intensity of GFP using a fluorescence microscope. The fluorescence intensity of GFP was quantified through Fiji by selecting one nematode head at a time in an image and measuring the area, integrated density and mean gray value. Using the calculation for the corrected total cell fluorescence (CTCF)—as described by McCloy et al., 2014—the fluorescence intensity was calculated using Excel to normalize against autofluorescence. Therefore, the expression level of LGG-1::GFP, SOD-1::GFP and SOD-3::GFP was examined in three independent experiments, each consisting of three independent groups (10 worms per group; *n* = 90).

### 2.8. Oxygen Consumption Rate of C. elegans

All experiments were performed on synchronized nematodes at day 2 of the adult stage. First, 24 h following irradiation, the worms were washed in S-basal medium to remove all traces of bacteria. Between 18 and 25 nematodes were placed in wells of the Seahorse XFe96 plate. The final volume in each well was 200 μL. For each condition and nematode strain, 3 wells (groups) were prepared. In addition, a minimum of 4 “blank” wells were made. They each contained 200 μL of sterile M9 medium. The measurement of the oxygen consumption rate in the irradiated and non-irradiated groups of *C. elegans* was performed using the Agilent Seahorse XFe96 Analyzer (Seahorse Bioscience, North Billerica, MA, USA). An average of the background-corrected OCR (pMol O_2_/min) between the different blank measurements was calculated, then the OCR measurements were normalized according to the number of individuals in each well. The mean and standard deviation of OCRs per nematode were calculated between the replicates for each condition using the Wave software. A variation is expected between wells. Therefore, each experiment was carried out in 3 wells. The results are expressed as a relative value with respect to the non-irradiated group.

### 2.9. Microdosimetry Calculations

Monte Carlo simulations based on the Geant4 10.07.p02 code [27,28] were performed to determine the specific energy per proton in the irradiated cell. The transport of primary particles and associated secondary electrons was carried out using Livermore models adapted to low-energy transport with a production threshold at 250 eV. The *C. elegans* were represented by a cylinder of liquid water, 50 µm in diameter, on the surface of a 4 µm thick polypropylene foil. The cell was modelled using a sphere of liquid water of 2.5 µm radius centered in the *C. elegans*. The primary particles were generated at the exit of the polypropylene foil to cover the entire surface of the cell. For this purpose, the physical characteristics of the primary particles (average energy, energy dispersion) were determined using the SRIM software [29].

### 2.10. Statistical Analysis

All data were expressed as mean ± Standard Error of the mean (SEM). The mean of fluorescence intensity normalized to background was calculated using the CTCF method and the samples consisted of 3 independent groups of worms (*n* = 90), obtained from 3 independent irradiation sessions. Asterisks indicate a significant difference in the independent *t*-test to the non-irradiated control and *p*-values of <0.05 were considered statistically significant. Fold-change was determined using the same number of samples described above. Asterisks indicate significance in a one-sample *t*-test against zero using the log2-transformed fold-change values and *p*-values of <0.05 were considered statistically significant. Histograms were generated using the Origin Pro Software. All statistical tests were performed using the SPSS Software.

## 3. Results

### 3.1. Monte Carlo Simulations for Dosimetry

**Figure 1:**
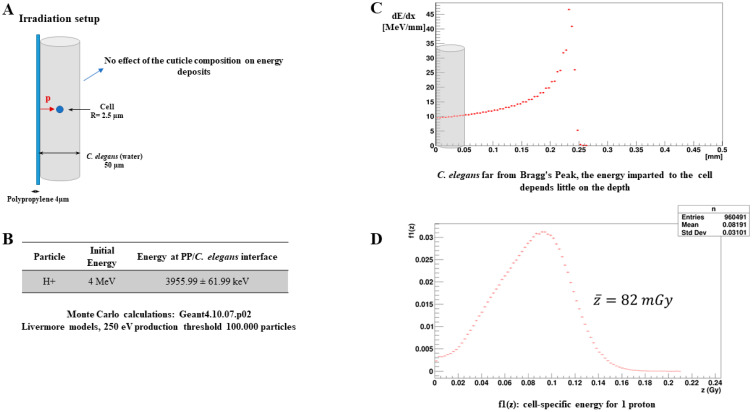
Microdosimetry calculations Monte Carlo simulations based on the Geant4 10.07.p02 code were performed to determine the specific energy per proton in the irradiated cell. (**A**). *C. elegans* were represented by a cylinder of liquid water 50 µm in diameter on the surface of a 4 µm thick polypropylene foil. The cell was modelled by a sphere of liquid water of 2.5 µm radius centered in *C. elegans*. The primary particles were generated at the exit of the polypropylene foil to cover the entire surface of the cell. For this purpose, the physical characteristics of the primary particles (average energy, energy dispersion) were determined with the SRIM software. (**B**). The transport of primary particles and associated secondary electrons was carried out using Livermore models adapted to low-energy transport with a production threshold at 250 eV. (**C**). *C. elegans* far from Bragg’s Peak, the energy imparted to the cell depends little on the depth. (**D**). The mean specific energy per proton in a cell is estimated to be 82 mGy.

The Monte Carlo simulations performed first verified the position of the *C. elegans* along the energy deposition profile (Figure 1C). Our results show that the worms were irradiated outside the Bragg’s peak in a zone where the energy deposit is constant with the depth. Thus, the specific energies calculated for the cell at the center of the worm are roughly the same as those for the other positions of the cell. The spectra in the imparted energy for one proton (Figure 1D) have been obtained for a sufficient number of primary particles, transported with adapted electromagnetic physical models (Figure 1B). The resulting mean specific energy per proton in the cell is estimated to be 82 mGy.

### 3.2. Protons Induce Oxidative Stress

Oxidative stress is considered as a fundamental process by which ionizing radiation inflicts damage to cells. The generation and persistence of radiation-induced oxidative stress result from cascading processes, passing from physical phenomena to biochemical and, finally, biological phenomena [30]. Cells are equipped with antioxidant systems that include antioxidant proteins. Among these, super oxide dismutase (or SOD) transforms superoxide anion O_2_^−^ into H_2_O_2_, while catalase transforms the hydrogen peroxide H_2_O_2_ thus produced into H_2_O and O_2_. These two types of enzymes are present everywhere in the cells and are found in particularly high levels in the mitochondria. However, the antioxidant defense can, in some instances, no longer keep up with the accumulation of ROS or can itself be damaged by them. ROS then accumulate dangerously in the cells, going so far as to damage the mitochondria and modify the production of energy, further increasing the mitochondrial production of the ROS in a vicious circle [31,32,33].

**Figure 2:**
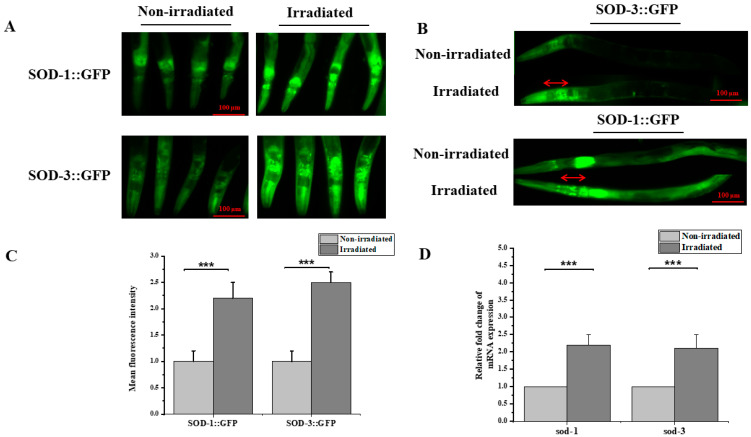
Proton microbeam irradiation induced SOD-1 and SOD-3 expression in the nerve ring region of *C. elegans*. (**A**) Relative epifluorescence images of the expression pattern in *C. elegans* reporter strain *sod-1:*:GFP and *sod-3*::GFP 24 h after exposure to 220 Gy protons in the head. (**B**) Absence of irradiation pattern in non-irradiation region of worm (head to tail orientation). (**C**) SOD-1::GFP and SOD-3::GFP fluorescence intensity assessed in vivo in the head of reporter strains *sod-1*::GFP and *sod-3*::GFP after 24 h of exposure to 220 Gy of proton radiation (independent *t*-test, ***: *p*-value < 0.001). (**D**) Relative fold change of mRNA levels of *sod-1* and *sod-3* were determined by RT-qPCR (one-sample *t*-test against zero, ***: *p*-value < 0.005).

In this study, we analyzed the post-irradiation oxidative stress in the nerve ring region of *C. elegans*. The head region of the *sod-1*::GFP and *sod-3*::GFP reporter strains was targeted with 2700 protons and the fluorescent signal in the irradiated zone was quantified 24 h after irradiation as an indicator of mitochondrial oxidative stress. The irradiated zone was a spot of 50 µm, with the nerve ring being the center of the deposition of 2700 protons. The total absorbed dose is estimated to be 220 Gy. In the irradiated region, the SOD-1::GFP and SOD-3::GFP expression at 24 h of irradiation increased significantly compared to the non-irradiated nematodes (Figure 2A–C). To further confirm these results, we decided to analyze the gene expression profiles of *sod-1* and *sod-3* in the irradiated region through RT-qPCR. A significant increase was observed for both *sod-1* and *sod-3* after 5 h (results not shown), reaching a 1.9-fold change 24 h after the exposure (Figure 2D). To the best of our knowledge, this is the first microbeam analyses of the *sod-1* and *sod-3* response after proton exposure in a localized region using living animals.

### 3.3. Increased Mitochondrial Copy Number

**Figure 3:**
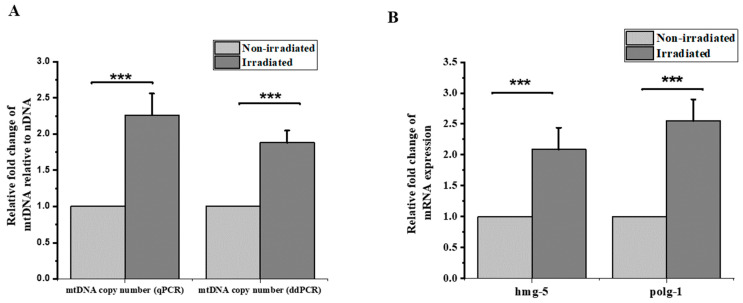
Proton irradiation induced an increase in the mtDNA copy number and mtDNA transcription. (**A**) Relative fold change in mtDNA copy number normalized to nDNA using qPCR and ddPCR (one-sample *t*-test against zero, *p*-value < 0.001). (**B**) Relative fold change of mRNA levels of *hmg-5* and *polg-1* as determined by RT-qPCR (one-sample *t*-test against zero, ***: *p*-value < 0.001).

Several studies have provided an indication that oxidative stress can induce variations in the mtDNA copy number. A mitochondrion contains multiple copies of its genome. The proper control of the mitochondrial DNA copy number is believed to be important for normal cell function. In this experiment, we quantified the mitochondrial DNA copy number in the heads of worms (ten heads per group) following exposure to 2700 protons through qPCR (~220 Gy). Our results show a significant increase in the mtDNA copy number, with a 2.1-fold change 24 h after irradiation compared to the non-irradiated control. These results were further validated using ddPCR technology for the absolute quantification of the mtDNA copy number normalized to nuclear DNA in the same condition of exposure. As described by Maremonti et al. (2020), ddPCR provides higher precision and greater sensitivity than qPCR for the detection of mtDNA copy number variation, as qPCR provides only a relative analysis because the quantification is based on the interpolation of a sample signal against a standard curve. The results obtained from the three independent experiments using ddPCR (each consisted of three independent groups) confirmed the same tendency provided by qPCR quantification, with a 1.8-fold increase in the mtDNA copy number normalized to nuclear DNA (Figure 3A).

Furthermore, recent studies revealed that the mtDNA copy number is directly regulated by putative mitochondrial nucleoid proteins in *C. elegans* [34,35]. In *C. elegans*, *hmg-5* (encoded by F45E4.9) is the ortholog of human TFAM, necessary for the transcription, replication and maintenance of the mtDNA copy number. In addition, mitochondrial DNA polymerase gamma (*polg-1*) is the sole DNA polymerase responsible for all replication and repair reactions within the mitochondria of mtDNA and its mutation, as well as its knock down, which resulted in a dramatic decrease in the mtDNA content in the *C. elegans* [36,37]. Based on these known mechanisms, we quantified the expression of *hmg-5* and *polg-1* through RT-qPCR. Interestingly, our data showed a 3-fold increase in the expression of *hmg-5* and a 2-fold increase in the expression of *polg-1* (Figure 3B). These results further confirm that the mtDNA replication machinery in the irradiated region is activated after proton irradiation.

### 3.4. Protons Induce Immediate Loss of Mitochondrial Membrane Potential

The mitochondrial polarization status directly affects mitochondrial function. Healthy mitochondria are polarized (negative inside) and sustain a highly charged membrane potential for full functionality [38]. Membrane potential is a key feature of mitochondria, as the loss of potential across the membrane is accompanied by a variety of cellular responses, including cytochrome c release, which is involved in apoptotic cell death. Stress-induced mitochondrial damage can cause a loss of mitochondrial membrane potential, leading to the mitochondria undergoing either fission or fusion.

Tetramethyl rhodamine ethyl ester (TMRE), a cationic fluorophore that accumulates electrophoretically in polarized mitochondria, enables the assessment of the mitochondrial membrane potential and, therefore, the mitochondrial function, allowing for changes in the membrane potential to be visualized very rapidly [38,39].

**Figure 4:**
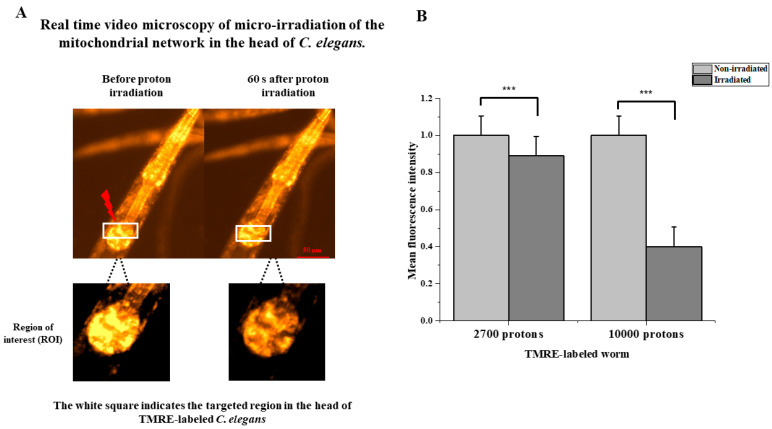
Protons induce instant dose-dependent mitochondrial membrane loss. (**A**) In vivo fluorescence analysis of mitochondrial membrane potential in N2 worms labeled with TMRE. Live imaging with fluorescence microscopy of targeted region revealed instant loss observed within seconds after irradiation. (**B**) Several worms were irradiated in the nerve ring with 2700 protons (*n* = 24) and 10,000 protons (*n* = 10). Quantitative analysis of fluorescence intensity in the irradiated region between *t* = 0 s and *t* = 3 min (paired *t*-test, ***: *p* < 0.005).

We observed the evolution of the membrane potential of the mitochondria during the irradiation through a proton beam, thanks to the TMRE marker. In these experiments, two doses were selected to detect any dose-dependent variations. A total of 2700 protons per point (*n* = 24) and 10,000 protons per point (*n* = 10) was deposited. Monitoring with an image every 10 s made it possible to visualize, in real time, the zone progressively impacted by the formation of the network. Figure 4A shows the monitoring of irradiation over 3 min, allowing the precise observation of the immediate depolarization of the mitochondrial network impacted by the pattern of the proton beam. In addition to the qualitative results, a quantitative study on the membrane potential of the mitochondria after irradiation with a proton beam was carried out. With the TMRE as a marker of the mitochondrial membrane potential, a decrease in the intensity of the labeling over time therefore reflects a decrease in the membrane potential. The intensity of the TMRE labeling was therefore measured. The results presented (Figure 4B) show a significant dose-dependent decrease in the fluorescence intensity, with a 10% decrease after irradiation with 2700 protons and up to a 60% decrease with 10,000 protons. The adjacent mitochondria of the directly impacted mitochondria were affected little or not at all by the depolarization phenomenon. This reflects that the loss of membrane potential occurs during direct impact with the proton beam and on the localized area.

### 3.5. Proton Irradiation Induced Autophagy

**Figure 5:**
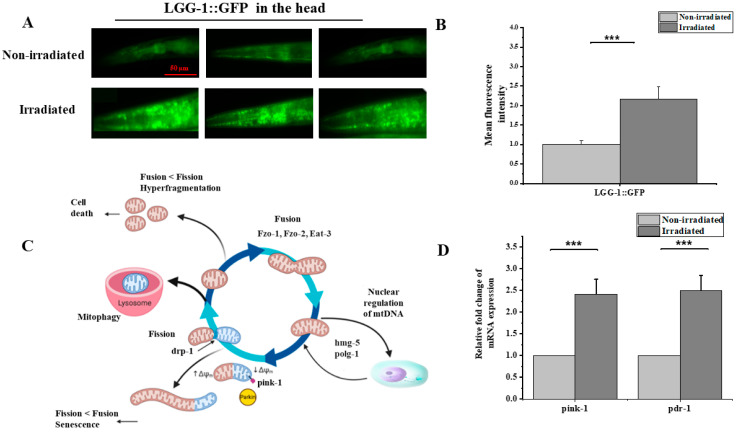
Proton microbeam irradiation induced autophagy in the nerve ring of *C. elegans* .(**A**) Relative epifluorescence images of the expression pattern in *C. elegans* reporter strain LGG-1::GFP 6-h after exposure to 220 Gy protons in the head. (**B**) LGG-1::GFP fluorescence intensity assessed in vivo in the head of *C. elegans* reporter LGG-1::GFP after 6 h of exposure to 220 Gy of proton radiation (independent *t*-test, ***: *p*-value < 0.01). (**C**) Schematic representation of programmed mitophagy, various events of mitophagy that work for the normal functioning of mitochondria by eliminating the damaged cell organelles and carrying out normal physiology. Adapted from [42] (**D**) Relative changes in mRNA levels of *pink-1* and *pdr-1* were determined by RT-qPCR (one-sample *t*-test against zero, ***: *p*-value < 0.005).

Mitochondria are very dynamic organelles. Their number and morphology mainly depend on the fusion and fission mechanisms whose actors are known in *C. elegans* (*fzo-1*, *fzo-2*, *eat-3* and *drp-1*, *pink-1*, *pdr-1*). When the mitochondrial damage is high, and often irreparable, it is necessary for the cell to set up another process to eliminate defective mitochondria (Figure 5C). Autophagy is one of the cellular degradation processes that has been conserved in all eukaryotic organisms. It has been proven that this degradation is selective and specifically targets cytoplasmic constituents and organelles such as mitochondria. Eukaryotic species have thus developed a specific degradation process that makes it possible to target mitochondria, called mitophagy. To be degraded by mitophagy, mitochondria must be labelled. Through the *pink-1*(*PTEN*-induced putative kinase 1)/*pdr-1* (ortholog of *PARKIN* in mammalians, Parkinson’s disease-related 1) protein pathway, the *pink-1* protein is recruited to the outer membrane of the mitochondria, phosphorylating both ubiquitin and the Parkin protein, and activating the latter. Parkin will then bind ubiquitin to the protein substrates of the outer membrane, and the complex formed is then recognized by the lgg-1 (*C. elegans* ortholog of mammalian LC3) protein anchored in the membranes of the phagophore, which allows the recruitment and sequestration of mitochondria within the phagophore [40].

The results showing the instant loss of the mitochondrial transmembrane potential, the anti-oxidative stress response and duplication in the mtDNA copy number raised our interest in investigating the impact of proton irradiation on the mitochondrial dynamics. We combined imaging observation and a genetic assessment of the transcript levels of *pink-1* and *pdr-1* using the RT-qPCR technique following exposure to 2700 protons in the head region. To detect autophagy, the LGG-1 (LC3/ATG8 homolog of *C. elegans*) GFP reporter strain DA2123 [*lgg-1p*::GFP::lgg-1 + *rol-6 (su1006)*] was used, as previously described [41]. DA2123 adults were irradiated with 2700 protons in the head region and cultured in the dark at 20 °C for 6 h with *E. coli.* We observed the GFP fluorescence through fluorescence microscopy during incubation, and the strongest fluorescence signal was observed at around 5 to 6 h (Figure 5A). We measured 30 worms (three independent groups of ten worms each) in each experiment and repeated this twice (total: *n* = 90). Consistent with previous studies, the fluorescence signal was 2.5-times stronger than that in the non-irradiated worms (Figure 5C). In addition, the gene expression analysis revealed a significant up-regulation of *pink-1* and *pdr-1* expression compared to the non-irradiated worms (Figure 5D). This result indicates proton-induced autophagy. Further experiments are needed to confirm selective mitophagy through the *pink-1 parkin* pathway.

### 3.6. Measurement of Oxygen Consumption Using SEAHORSE XFe96 Analyzer after Irradiation

The rate of oxygen consumption is a vital marker for indicating healthy mitochondrial function, reflecting the ROS production and metabolic activity of the cell. To foster our understanding of the mechanism by which protons induce mitochondrial dysfunction, we measured the oxygen consumption in the irradiated *C. elegans*. Despite the advantage of microbeam irradiation, mainly in understanding the tissue-specific response to ionizing radiation compared to whole-body exposure, the limited number of irradiated worms represented a challenge to these experiments. As it was not possible to measure the oxygen consumption rate in the head exclusively with the available methods, we analyzed the whole-body consumption using the Seahorse XFe96 Analyzer [43].

**Figure 6:**
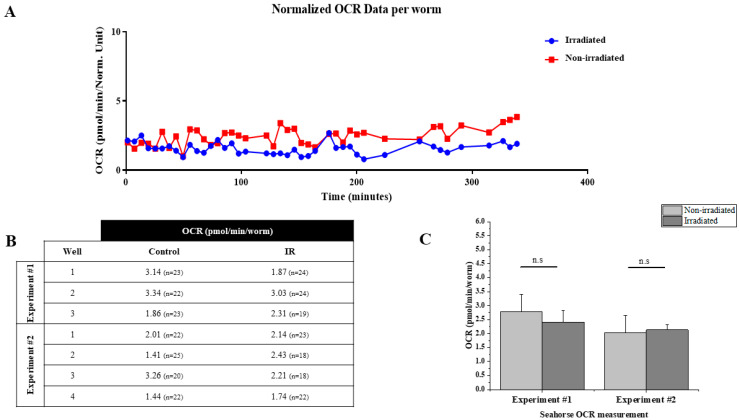
Micro-irradiation of nerve ring did not affect the whole-body oxygen consumption rate (OCR) (**A**) OCR measurement of irradiated and control worms using Agilent Seahorse XFe96 Analyzers. Normalized OCR data per worm after each measurement is generated by Wave 2.6 software. (**B**) Summary of data from 2 independent experiments. Experiment #1 *n* = 67 and Experiment #2 *n* = 81 worms. (**C**) Relative change of OCR in each measurement. *n*.s indicates the absence of significant change in OCR between irradiated and non-irradiated worms. Asterisks indicate significant difference to non-irradiated control at 24 h (independent *t*-test, n.s. means not-significant: *p*-value = 0.15).

The Seahorse XFe96 Analyzer (Seahorse Bioscience, North Billerica, MA, USA) is a device that measures the rate of oxygen consumption (OCR: Oxygen Rate Consumption) and the rate of extracellular acidification (ECAR: Extracellular Acidification Rate) on living cells in a 96 plate well. This tool allows the real-time calculation of OCRs and ECARs. It thus offers the possibility of studying key cellular functions, such as mitochondrial respiration and glycolysis. The Seahorse XFe96 was initially created to work on cells. Since then, it has been used in many fields, including senescence, oncology, cellular metabolism, mitochondrial diseases and on whole organisms. Oxygen consumption was measured using this tool for the first time in *C. elegans* by Dancy et al. in 2013. It was then used for various studies on mitochondrial functioning (Andreux et al., 2014; Luz et al., 2015) and the relationship between mitochondria and longevity in *C. elegans* (Mouchiroud et al., 2013). However, none of these works have studied—on a large scale and on a large number of individuals—the impact of ionizing radiation on the mitochondrial respiratory chain in *C. elegans*. In this work, the measurement of the basal oxygen consumption on the organism *C. elegans* using the Seahorse XFe96 Analyzer on the wild strain N2 is described. Two independent measurements were performed. Each comprised three independent groups of 18 to 25 irradiated adult hermaphrodites (Figure 6B). The oxygen consumption rate was adjusted to the number of individuals and then compared to the non-irradiated controls (Figure 6A). Our data show that the micro-irradiation of worms’ heads does not significantly affect the whole-body oxygen consumption rate despite the slight decrease (Figure 6C). Whether this result reflects the existence of a compensation mechanism allowing the maintenance of stable oxygen consumption after localized irradiation or that the effect on oxygen consumption is diluted by the whole-body measurement remains a question and requires further analysis.

## 4. Discussion

It is perhaps surprising that over a century has elapsed since the discovery of ionizing radiation; however, the mechanisms by which exposure to radiation induces damage to cells remain poorly understood. This understanding is indispensable to ultimately improving the efficiency and safety of radiotherapy, especially when used to treat patients with brain cancers. Epidemiological data, supplemented with experimental work, have confirmed that radiation induces molecular and cellular modification in the brain [3,4,44]. The development of increasingly sensitive and specific techniques has made it possible to better understand the molecular basis of the undesirable side effects. The long-believed dogma was centered around DNA damage being the only pathway that leads to radiation damage. However, the discovery of non-targeted phenomena, such as the bystander effects on cells that have not directly been exposed to radiation and the risks associated with low doses of radiation exposure, have challenged this dogma [3,17,18].

The data inferred from studies investigating the impact of ionizing radiation on mitochondrial function and structure raised our interests to investigate the impact of proton irradiation on mitochondrial function in the nerve ring region of *C. elegans* [18]. *C. elegans* are a well-established research tool suitable for radiobiological studies—as discussed elsewhere—and its implementation in microbeam studies is rising [20,21]. Although each piece of information from microbeam studies is valuable, an essential challenge in microbeam experiments is increasing its efficiency as the ability to perform microbeam experiments is limited and cannot be performed frequently. The proposed method of microbeam irradiation with an exact number of particles allows us to irradiate a specific region of the body to analyze the immediate and prolonged effects of irradiation in several living animals. In this work, we irradiated a limited region in the head of adult *C. elegans*, with the nerve ring being the center of deposition, which is considered to be the worm’s brain [45].

In radiotherapy, depending on the type of radiation used and the cancer being treated, the human brain is exposed to up to 60 Gy, which is considered a high-dose irradiation [46]. However, it is well-known that species respond differently to ionizing radiation. Humans and mice (half-lethal dose -LD_50_- of 4 to 7 Gy) are more sensitive to radiation than some very radio-resistant animals, such as *Milnesium tardigradum* (tardigrades, LD_50_ of 5000 Gy), *Adineta vaga* (tetraploid rotifer, LD_10_ of 1200 Gy) and *C. elegans* (LD_50_ of 3500 Gy) [47]. Radiosensitivity also varies from one tissue to another within the same species, with mitotic cells being more sensitive. Non-dividing cells, such as neurons, are less sensitive than mitotic cells, such as gametes, and are damaged by high doses. Given the high radioresistance of *C. elegans*, the mitochondrial function in the central nervous system of worms was assessed after irradiation with 220 Gy of protons, which fell within the margin of high-dose irradiation for *C. elegans.* In this study, we show the first results of 650 individually irradiated *C. elegans* in the head region on a MIRCOM microbeam.

Oxidative stress is a well-documented effect of incident radiation when it hits the cell. Under normal conditions, mitochondrial respiration reduces up to 97% of oxygen, and the remaining percentage of incompletely reduced oxygen is transformed to superoxide radical anion (O_2_^−^) and then transformed into H_2_O_2_ by the mitochondrial isoforms SOD-2 and SOD-3. In the case of excessive production of O_2_^−^, it may also leak into the cytosol to become the substrate for the cytosolic Cu, Zn-sod (SOD-1) [48]. Thus, understanding the activity of SOD-1 and SOD-3 may reflect the oxidative status in the cell. The fluorescent signal was observed in the head with a significant increase 24 h after irradiation, thus reflecting the anti-oxidative response by inducing the expression of SOD-1 and SOD-3. However, no clear induction was observed in the non-irradiated tissue of *C. elegans*. This result is similar to previous observations in mice and *C. elegans*, indicating that radiation induces the up-regulation of SOD-1 and SOD-3 [48,49,50]. The increased expression observed within 24 h after irradiation implies the continuous formation and accumulation of O_2_^−^ following the exposure, considering that the SOD-1 and SOD-3 response was about 3-fold higher compared to the non-irradiated worms. These observations are consistent with the LET model for radiolysis radical formation [51] and with previous studies showing that the scavenging system plays a key role in radiation tolerance [52]. As discussed above, *C. elegans* are considered to be among the highly radio-resistant animal models. Therefore, we suggest that exposure to proton radiation may induce the accumulation of O_2_^−^ inside the mitochondria and the cytosol and stimulate the antioxidant response. Despite the fact that further studies are still needed to understand the changing redox balance for long durations and whether this effect is partially or completely reversed, even a minor change can result in a substantial alteration; for example, in terms of metabolism, cell proliferation and host defense [53]. The changes in the redox status of the nematodes could cause significant oxidative damages and affect the mitochondrial function in the targeted cells [54]. 

Furthermore, there is an unequivocal link between the mtDNA copy number and mitochondrial function. Variations in the mtDNA copy number are often used as a readout to measure radiation response for mitochondrial function. We hypothesized that the quantification of mtDNA may be an indicator of the mitochondrial response to proton exposure, especially after oxidative stress. Our results showed a 2-fold increase in the mtDNA copy number with the activation of mtDNA replication machinery, characterized by the up-regulation of the *hmg-5* and *polg-1* genes, which are both involved in the regulation of the mtDNA level. These results agree with previous studies that revealed an increase in the mitochondrial DNA copy number after ionizing irradiation in different models, in vitro and in vivo, and reviewed by Kam et al. until 2013 [17]. Recently, several studies have also reported an increase in the mitochondrial copy number following irradiation. Maremonti. et al. (2020), using *C. elegans*, revealed that mtDNA increased in a dose-dependent manner in response to chronic gamma radiation. However, a significant increase in the mtDNA copy number was only evident for dose rates as high as ∼1 Gy.hr^−^ [1] provided for an extended period of time (24–72 h) [25]. In addition, the mtDNA copy number doubles in rat brain regions (the hippocampus, cortex and cerebellum) within 6–24 h of radiation time (5 Gy, X-rays) [55]. Moreover, dogs irradiated in the lungs with 18 Gy of X-rays exhibited a 2.2-fold increase 8 weeks after irradiation in the targeted tissues [56]. The same trend of increases was found in bank voles (*Myodes glareolus*) collected from the Chernobyl exclusion zone (CEZ), where animals are exposed to elevated levels of radionuclides, and from uncontaminated areas within the CEZ and elsewhere in Ukraine. The brains of bank voles within the CEZ had high mtDNA copy numbers and high mtDNA damage compared to the control group [57]. Our results indicate that the activation of mitochondrial biogenesis with mtDNA replication in the irradiated brain is triggered within 24 h after proton exposure, resulting in an increase in mtDNA content in the targeted region, consisting mostly of neurons. This assumption is consistent with previous results obtained in vitro (neurons) and in vivo (mouse and rat brain)—discussed above—and may be accentuated by the high energy demands of neurons, which lead to the replenishment of dwindling energy levels. In addition to the compensatory mechanism that leads to an increased mtDNA copy number, the latter was found to be correlated with oxidative stress levels in human leukocytes [17]. Thus, an increase in the mitochondrial content could be a transient gain that may later strain the cells as more resources are needed to cope with the subsequent increase in ROS.

Furthermore, in light of these findings, we analyzed the mitochondrial membrane potential following targeted irradiation. The study of the membrane potential using the TMRE marker has shown a local depolarization of mitochondrial membranes due to the targeted passage of protons in the mitochondrial network of cells. This depolarization is dose-dependent, with a slight 10% decrease at 2700 protons per point (*n* = 24) and a sharp decrease of around 60% with 10,000 protons per point (*n* = 10). This result is consistent with the work of Walsh et al., in 2017 on the AIFIRA microbeam in Bordeaux. This team showed comparable results in the in vitro study of the mitochondrial membrane potential using the same type of charged particles with the same energy [38]. In addition, previous studies on cells on the MIRCOM microbeam made it possible to validate the TMRE marker by inducing a 25% depolarization on two different types of cell cultures (data not shown). Walsh’s team also showed a re-localization of the TMRE in the cytoplasm, as well as a depolarization wave phenomenon. This phenomenon results in a localized drop in the targeted area, followed by a generalized drop in the intensity of the TMRE fluorescence signal. This is indicative of the depolarization of mitochondria, step by step, until it affects the entire mitochondrial network. These reactions could not be observed in our study; however, it is important to note that the cells used in the research by Walsh et al. are MCF-7 cells from a breast adenocarcinoma. The fact that the cells do not come from the same type of tissue and are also cancer cells makes them very different from the type of irradiation carried out on living animals. This might not be the only reason for the observed differences. Indeed, the phenomenon of photobleaching could be too important on the images that we observed and would prevent us from detecting the phenomenon of a wave of depolarization or re-localization of the TMRE. Furthermore, our conclusions concerning the localized depolarization of the mitochondrial network on the passage of protons agree with the conclusions of the work of Walsh’s team, which worked on other cell types and with similar charged particles (protons) and different ones (ions carbon). Our results are therefore in line with the knowledge already acquired by the scientific community in the field of the irradiation of mitochondria and open the way to new similar studies with other types of radiation or cells. To the best of our knowledge, this is the first study of mitochondrial membrane potential following microbeam irradiation in a living animal. The drop in intensity of the TMRE is of the order of 10% after the passage of the beam of 2700 protons in a localized zone around the impact and of 60% at 10,000 protons. However, this quantitative result deserves the implementation of larger studies, although it remains complicated in the field of targeted microbeam irradiation to carry out large-scale studies due to the complexity of the experiments. This problem therefore recurs in most studies, such as in that of Walsh et al. in 2017, where a group of 20 cells was used for the statistical analysis of the decrease in intensity of the TMRE 10 min after irradiation [38]. Furthermore, it would have been interesting to be able to observe the phenomenon of the repolarization of the mitochondrial membranes, which occurs if the functions of the mitochondria are not too damaged. In theory, this only occurs after several hours (between 12 and 48 h) after the first phenomenon of depolarization caused by an external stress. However, this study would have been too complex to implement on the platform of our study.

Autophagy is one of the mechanisms by which non-functioning mitochondria are degraded [41]. It was previously reported that autophagy is induced by ROS after 7 h in *C. elegans* intestinal cells using the lgg-1p::GFP::lgg-1 reporter method [41]. Using this method, Yamasaki et al. (2021) reported the induction of autophagy through radiation (carbon particles) and found that autophagy was also induced after 7 h, mainly in the pharynx (head), intestinal tract (anterior body) and motor neurons along the body-wall muscles. In line with their findings, the irradiation of the nerve ring with 2700 protons resulted in an increase in autophagy, observed 5 h after irradiation, accompanied by the up-regulation of *pink-1* and *pdr-1* genes expression. This suggests that the quality control pathways for mitochondria are locally activated in response to irradiation as no clear induction was observed outside the targeted region in the head. In *C. elegans*, gamma radiation induced the expression of *pink-1* [58]. In addition, Bess et al. (2012) reported that mitochondrial dynamics and autophagy aid in the removal of persistent mitochondrial damage in *C. elegans* [59]. 

Finally, we hypothesized that radiation may induce metabolic changes following mitochondrial damage. Mitochondria rely on the electron transport chain (ETC) to produce energy as ATP. The ETC is composed of respiratory chain complexes I–IV, which transfer electrons until they finally reduce oxygen to form water, causing oxygen consumption [60]. It is widely accepted that the measurement of the oxygen consumption rate (OCR) of a living organism, as an indicator of OXPHOS activity, provides an indirect method to evaluate the energy metabolism of mitochondria [60,61,62]. Using the available techniques, it was not possible for us to evaluate the oxygen consumption rate only in the irradiated region. We analyzed the OCR of the whole organism, and we found the slight decrease in OCR after 24 h to be not statistically significant. Several studies previously reported that radiation exposure may influence the oxygen consumption rate, mainly in cancer cells [63,64,65]. Further investigations are still needed for us to understand whether the maintenance of a normal OCR in the nematode is due to the compensation mechanism or dilution effect of the localized irradiation impact in the whole-body measurement of the OCR.

## 5. Conclusions and Perspectives

Collectively, our results indicate global damage in mitochondrial function after exposure to proton radiation in a targeted region in the head, where most neurons are located. Normal mitochondrial function is critical for neurons, but also for other cell types in the nervous system. Mitochondrial dysfunction can alter the function of other cell types in the nervous system known to be implicated in several neurological diseases, such as astrocytes and glial cells. This work examined several factors related to radiation-induced mitochondrial dysfunction. These factors include variations in the mtDNA level, oxidative stress, autophagy and mitochondrial respiration. Together, mitochondrial dysfunction and the subsequent oxidative stress are implicated in the radiation-induced damage of irradiated cells. As most *C. elegans* neurons are concentrated in the targeted region, it is envisioned to follow this work by investigating the radiation-induced behavioral change of *C. elegans.* Through an improved understanding of the mitochondria-dependent mechanisms of radiation-induced dysfunction, potential therapeutic targets can be developed to assist in the prevention and treatment of radiation-induced damage.

## Data Availability

The data that support the findings of this study are available from the corresponding author upon reasonable request.
